# Postsurgical morbidity and mortality favorably informs deep brain stimulation for new indications including schizophrenia and schizoaffective disorder

**DOI:** 10.3389/fsurg.2023.958452

**Published:** 2023-03-30

**Authors:** Judith M. Gault, Patrick Hosokawa, Daniel Kramer, Elyn R. Saks, Paul S. Appelbaum, John A. Thompson, Ann Olincy, Nicola Cascella, Akira Sawa, Wayne Goodman, Nidal Moukaddam, Sameer A. Sheth, William S. Anderson, Rachel A. Davis

**Affiliations:** ^1^ Department of Neurosurgery, University of Colorado Anschutz Medical Campus, Aurora, CO, United States; ^2^ Department of Psychiatry, University of Colorado Anschutz Medical Campus, Aurora, CO, United States; ^3^ The Law School, University of Southern California, Los Angeles, CA, United States; ^4^ Department of Psychiatry, Columbia University, New York, Ny, United States Of America; ^5^ VA Eastern Colorado Medical Center, Aurora, CO, United States; ^6^ Department of Psychiatry, Johns Hopkins University, Baltimore, MD, United States; ^7^ Department of Psychiatry, Baylor College of Medicine, Houston, TX, United States; ^8^ Department of Neurosurgery, Baylor College of Medicine, Houston, TX, United States; ^9^ Department of Neurosurgery, Johns Hopkins University, Baltimore, MD, United States

**Keywords:** deep brain stimulation, surgical disparities, surgical risk, surgical morbidity, surgical mortality, schizophrenia, schizoaffective disorder, Parkinson disease

## Abstract

**Background:**

Deep brain stimulation (DBS) shows promise for new indications like treatment-refractory schizophrenia in early clinical trials. In the first DBS clinical trial for treatment refractory schizophrenia, despite promising results in treating psychosis, one of the eight subjects experienced both a symptomatic hemorrhage and an infection requiring device removal. Now, ethical concerns about higher surgical risk in schizophrenia/schizoaffective disorder (SZ/SAD) are impacting clinical trial progress. However, insufficient cases preclude conclusions regarding DBS risk in SZ/SAD. Therefore, we directly compare adverse surgical outcomes for all surgical procedures between SZ/SAD and Parkinson's disease (PD) cases to infer relative surgical risk relevant to gauging DBS risks in subjects with SZ/SAD.

**Design:**

In the primary analysis, we used browser-based statistical analysis software, TriNetX Live (trinetx.com TriNetX LLC, Cambridge, MA), for Measures of Association using the Z-test. Postsurgical morbidity and mortality after matching for ethnicity, over 39 risk factors, and 19 CPT 1003143 coded surgical procedures from over 35,000 electronic medical records, over 19 years, from 48 United States health care organizations (HCOs) through the TriNetX Research Network™. TriNetXis a global, federated, web-based health research network providing access and statistical analysis of aggregate counts of deidentified EMR data. Diagnoses were based on ICD-10 codes. In the final analysis, logistic regression was used to determine relative frequencies of outcomes among 21 diagnostic groups/cohorts being treated with or considered for DBS and 3 control cohorts.

**Results:**

Postsurgical mortality was 1.01–4.11% lower in SZ/SAD compared to the matched PD cohort at 1 month and 1 year after any surgery, while morbidity was 1.91–2.73% higher and associated with postsurgical noncompliance with medical treatment. Hemorrhages and infections were not increased. Across the 21 cohorts compared, PD and SZ/SAD were among eight cohorts with fewer surgeries, nine cohorts with higher postsurgical morbidity, and fifteen cohorts within the control-group range for 1-month postsurgical mortality.

**Conclusions:**

Given that the subjects with SZ or SAD, along with most other diagnostic groups examined, had lower postsurgical mortality than PD subjects, it is reasonable to apply existing ethical and clinical guidelines to identify appropriate surgical candidates for inclusion of these patient populations in DBS clinical trials.

## Introduction

Deep brain stimulation (DBS) is revolutionizing the treatment of some neurologic and psychiatric brain disorders *via* identification and modulation of electrophysiological aberrancies within relevant circuits (2, 3). It is important that advanced DBS neurotechnology be accessible for new therapeutic indications and that ethical concerns about surgical interventions be considered in the context of severely disabling, treatment-refractory conditions like schizophrenia and schizoaffective disorder (SZ/SAD) (4). DBS is a United States Food and Drug Administration (FDA)-approved, standard-of-care neurosurgical intervention for treatment-refractory Parkinson's disease (PD), essential tremor (ET), and epilepsy. DBS also has FDA humanitarian device exemption (HDE) approval for treatment-refractory obsessive-compulsive disorder (OCD) and dystonia. Advances in DBS therapeutics have been accelerated with the development of segmented DBS leads that allow precise steering of current to maximize therapeutic options while minimizing side-effects, as well as DBS devices that can both stimulate and record electrophysiological activity (2, 5). Elucidation of underlying electrophysiological mechanisms of these treatment-refractory conditions is expected to dramatically improve targeting and optimize treatment, for example by guiding the development of closed loop or adaptive DBS.

As enthusiasm for DBS has grown, so has optimism about investigating DBS therapy for other treatment-refractory disorders affecting neural circuit including but not limited to obesity, traumatic brain injury (TBI), tinnitus (TIN), Alzheimer's disease (AD), multiple sclerosis (MS), Central Nervous System atrophy disorders (CNS atrophy), cerebral palsy (CP), chronic pain (CHP), and chronic spinal pain (SP) (6–9). Neuropsychiatric disorders including anorexia nervosa, substance use disorders, Tourette's syndrome (TS), autism spectrum disorder (autism), recurrent major depressive disorder (MDD), bipolar disorder (BP), and schizophrenia/schizoaffective disorder (SZ/SAD) are also being treated experimentally with DBS in clinical trials (1, 5, 10). However, enrollment into clinical trials for treatment-refractory SZ has been challenging, and one study closed due to difficulty enrolling patients (4). Overprotection of these vulnerable individuals related to concerns about surgical risk may be contributing to low participation in clinical trials (11–14).

SZ/SAD remains a severely disabling disorder for the majority of patients, as demonstrated by a low competitive employment rate of only 10%–20%, a 4.5-fold increased suicide rate, and 12–18.7-year reduced life expectancy relative to the general population (15–21). Despite antipsychotic adherence, 42%–60% of patients ultimately relapse and 10%–25% are treatment refractory (22–27). Modulation of relevant circuitry by antipsychotics is tied to and limited by their antagonistic action at dopamine D2 receptors (28). Although DBS will not be appropriate for the majority of subjects with treatment-refractory schizophrenia, and there are only reports of DBS in 10 subjects to date, for some treatment-refractory subjects tested, DBS is more potent at therapeutically modulating psychosis-related circuits of interest, and one treatment-refractory subject experienced acute, immediate alleviation of auditory hallucinations that was sustained for over a year (1, 29, 30). Clearly, larger clinical trials are warranted to determine DBS effectiveness for subjects with treatment-refractory schizophrenia and schizoaffective disorder.

The extant literature demonstrates increased morbidity (OR 0.9–15) and mortality (odds ratio, OR = 1.11–2.70) in patients with schizophrenia/schizoaffective disorder (SZ/SAD) compared to populations without mental illness in studies that included surgeries for breast cancer, appendectomy, acute coronary syndrome, total joint arthroplasty, and a large retrospective review of all major surgeries combined (31–43). Similarly, subjects with PD also experienced increased frequencies of surgical adverse events compared to populations without PD, including more colorectal surgical morbidity (+44%), hip arthroplasty complications (+65%), major complications (OR =  1.74–2.98) and mortality (+0.5%–5.5%) for spinal surgery (44–49). To inform ethical and clinical guidelines for inclusion in DBS clinical trials, it is important to directly compare the frequency of adverse surgical outcomes in patients with SZ/SAD to subjects with PD, the most common indication for DBS surgery.

The perception of surgical risk for people with severe psychiatric conditions like SZ/SAD and for neurosurgical interventions may be inflated because of historically high mortality rates of 15% for frontal lobotomies and 10% for early DBS before advanced stereotactic neurosurgery (50, 51). In addition, the vulnerability of subjects with psychosis may lead to their overprotection and overly paternalistic and exclusionary behavior even with established surgical procedures (11, 52). However, overprotection and a falsely heightened perception of surgical risk negatively impacts all three fundamental principles of the Belmont Report for the conduct of clinical trials: (1) respect for persons by raising the threshold for capacity to consent; (2) beneficence (and the related principle of non-maleficence the duty to avoid harm), by skewing the perceived risk-benefit ratio; and (3) justice, by decreasing accessibility to established and experimental surgical interventions (52, 53). As a result, patients with SZ who are competent to make their own decisions about entering a DBS trial and desire to do so may be denied the opportunity to exercise their choice.

DBS surgery is considered a minimally invasive and largely reversible intracranial surgery that is often conducted in stages. First, stereotactic surgery is used to precisely implant thin electrodes (leads) that deliver low levels of electricity to therapeutically modulate disease-specific brain circuits. Second, the neurostimulators and extension cables are inserted and connected under the skin. In DBS clinical trials for new indications, where benefits are largely unknown, applying the principle of beneficence relies heavily on weighing the likelihood that the hoped-for outcome will occur against the morbidity associated with the disease itself and the estimated risk of adverse outcomes. The desired outcomes here are a reduction of symptoms, an increase in functional capacity, and a decrease in related co-morbidities, including early mortality. These hoped-for benefits, invoking the principle of nonmaleficence, must be weighed against the likelihood of harms arising from the surgery. The best estimates of stimulation, device, and surgical risks are based on DBS performed as standard-of-care treatment in patients with PD (Table 1) (54). The most common surgical risks are mortality (0%–0.8%), intracranial hemorrhage (0%–7.5%), and infection (2.6%–10.7%) (54). However, these estimates of risk for new indications do not consider potential diagnosis-specific surgical risks, and, in the first DBS clinical trial for schizophrenia, despite promising results in treating psychosis, one of the eight subjects experienced both a symptomatic hemorrhage and an infection requiring device removal (1). However, surgical risk estimates based only on these few DBS cases are not accurate, and diagnosis-specific surgical risk must be assessed in a larger sample.

**Table 1:** Beneficence/non maleficence deep brain stimulation.

PD/ET DBS Adverse **E**vent**s (AE)**	Frequency
**DBS Tremor study *n* = 424**	
Patient with ≥1 device/surgery AE (i.e. 7% postoperative pain)	28%
Patients ≥ 1 stimulation AE (i.e. paresthesia 33%)	40%
***Death*** *, DBS-related*	*0.7%* *
**DBS PD study *n* = 160**	
Patients ≥ 1 device AE	96.3%
Stimulation adverse events	51.9%
Device adverse events	36.9%
Ongoing stimulation adverse events	22.5%
Ongoing device adverse events	10.0%
Stimulation serious adverse events	9.4%
Device serious adverse events	17.5%
Serious ongoing stimulation AE	3.1%
Serious ongoing device AE	6.3%
Infection	10.6%*
Paresis/asthenia	10.0%
Hemiplegia/hemiparesis	8.1%
Intracranial hemorrhage	7.5%*
***Death*** *, DBS-related*	*0.6%* *

* Life threatening; (Medtronic 2015).

Here, we characterize relative surgical safety for all surgical procedures among brain disorders where DBS is investigational, like SZ/SAD, and disorders where DBS is established, like Parkinson's disease (PD), to determine whether the risk for adverse events is higher for some diagnostic groups. Based on reports of increased surgical adverse events in both subjects with SZ/SAD and PD, we hypothesized that adverse postsurgical outcomes are similar in subjects with SZ/SAD compared to those with PD. In the primary analysis, we matched PD and SZ/SAD cohorts for over 39 pre-surgical factors including smoking, drug and alcohol dependence, age, and obesity that impact life expectancy, to allow direct comparisons of postsurgical morbidity and mortality for all surgeries combined. Then we determined how postsurgical morbidity and mortality for all surgeries compares to DBS-related neurostimulation surgeries in four diagnostic cohorts where DBS has FDA approval and is often used: PD, ET, DYS, and epilepsy. Relative frequencies of presurgical risk factors and 1-year post- DBS surgical morbidity and mortality was determined among PD, ET, DYS and epilepsy DBS cases. Finally, we characterized relative frequencies of all surgical procedures combined, pre-surgical risk factors, postsurgical morbidity, and mortality among 21 DBS-related diagnostic cohorts and 3 control groups. Our goal was to determine if surgical disparities and risk are higher, lower, or similar for people with SZ/SAD relative to other diagnostic groups/cohorts being treated with or considered for DBS.

## Materials and methods

**Figure 1:**
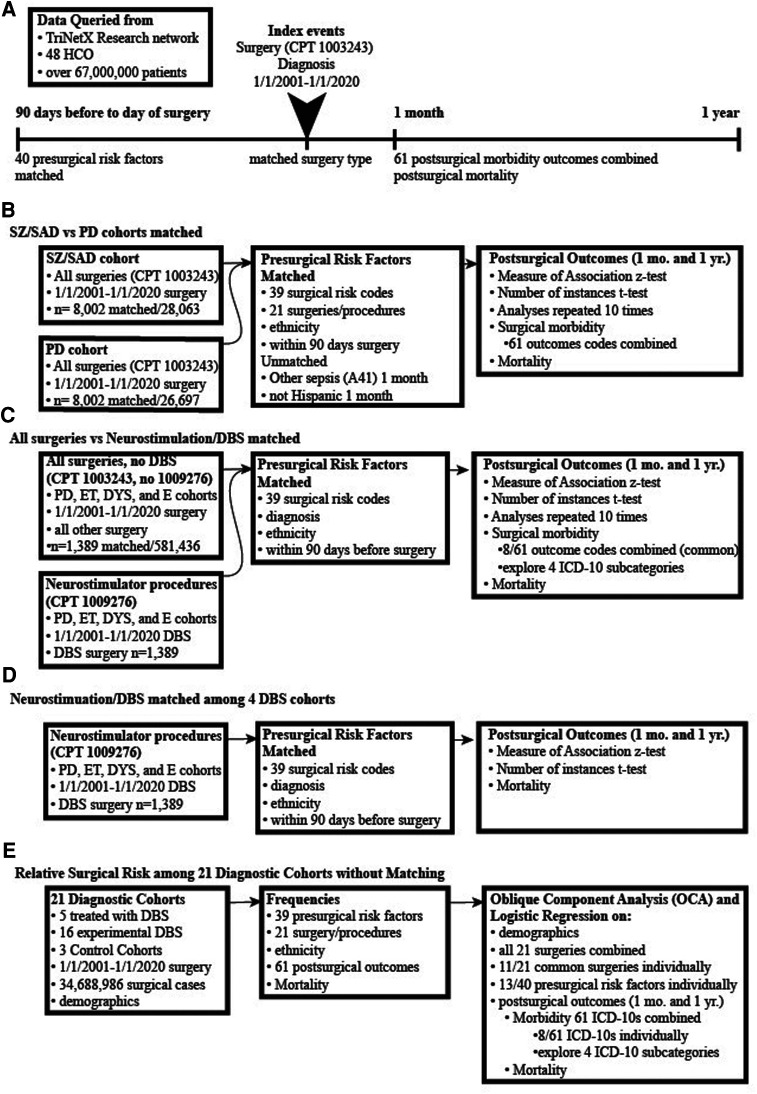
Pipelines of surgical data collection and analysis. (**A**) Cohorts were generated by database queries that included having a surgical procedure and diagnoses of interest (both were the index event). Matching occurred between factors occurring 90 days to the day of surgery. The postsurgical outcomes were analyzed at 1 month and 1 year. (**B**) Surgical outcomes of subjects diagnosed with either schizophrenia or schizoaffective disorder (SZ/SAD) were compared with subjects with Parkinson's disease (PD). The SZ/SAD and PD cohorts were matched for surgical risk factors and type of surgical procedures. (**C**) Subjects with a PD, essential tremor, dystonia, or epilepsy diagnosis were split into two cohorts, one with neurostimulation surgical procedures or with all other surgeries combined. Cohorts were matched for presurgical factors and diagnoses to determine the relative frequencies of postsurgical morbidity and mortality. (**D**) Matched analyses of DBS postsurgical (after 1-month and 1- year) morbidity and mortality outcomes in PD, epilepsy, essential tremor, and dystonia where compared. (**E**) The frequencies of surgery, presurgical factors and postsurgical outcomes were tabulated for 21 unmatched diagnostic cohorts and 3 controls cohorts.

This study is IRB approved without the requirement for patient consent (IRB#18-2885). We queried patient data retrospectively from electronic medical records (EMR) using three clinical/claim coding types: (1) current procedural terminology for surgery (CPT 1003143 is a parent term for all surgical procedures organized within CPT coding); (2) medication codes; and (3) International Classification of Diseases, Tenth Revision, Clinical Modification (ICD-10 codes) (Figure 1). All surgical procedures under the parent term “Surgery” CPT 1003143 were included in this analysis including all 19 surgical procedure types organized directly under Surgery, CPT 1003143, were matched in the primary analysis. This included general surgical procedures, reproductive system procedures, operating microscope procedures, intersex surgery, and surgical procedures on the integumentary system, musculoskeletal system, respiratory system, cardiovascular system, hemic and lymphatic systems, mediastinum and diaphragm systems, digestive system, male genital system, female genital system, maternity care and delivery, endocrine system, nervous system, eye and ocular adnexa, and auditory system. We obtained data from TriNetX Research Network™ (trinetx.com), a global, federated, web-based health research network providing access and statistical analysis of aggregate counts of deidentified EMR data from the Research Network of up to 48 health care organizations (HCOs). Each diagnostic cohort included patients who met the following two criteria: received one of the 21 diagnoses of interest and received any surgery from January 01, 2001, to January 01, 2020, (Figure 1A).

**Figure 2:**
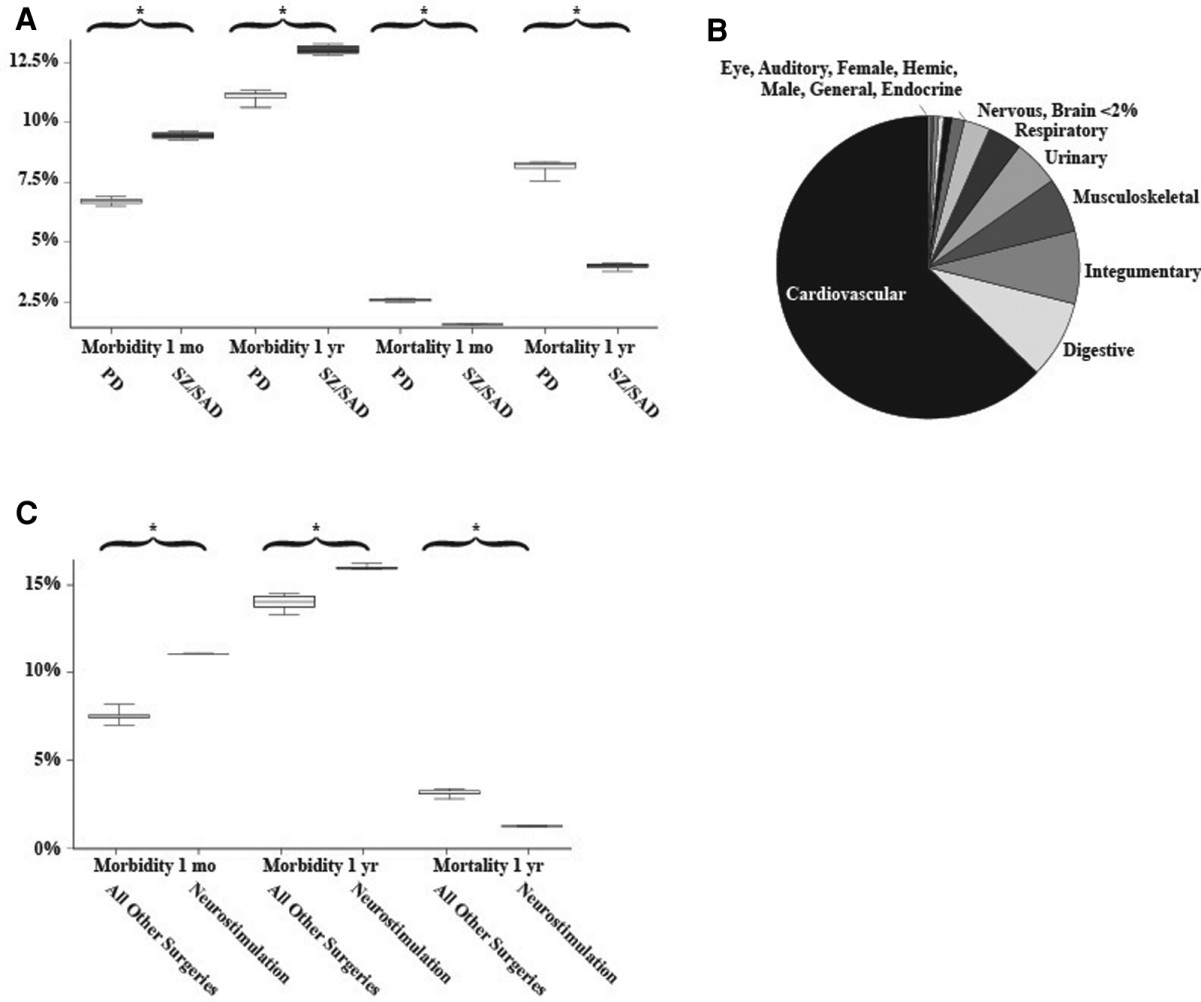
Matched analyses. (**A**) Frequencies of postsurgical morbidity and mortality between matched Parkinson's disease and schizophrenia/schizoaffective disorder cohorts (*n* = 15,354 surgical cases/cohort). (**B**) The relative proportion of 14 of the common surgical procedures examined. Microscope, Maternity, Diaphragm, Intersex, and Reproductive surgeries made up <0.2% of the total. (**C**) Comparison of postsurgical morbidity and mortality between all surgeries (without neurostimulation/DBS) and only neurostimulation/DBS surgeries (grey). 1,389-4,787 surgical cases among those with Parkinson disease, essential tremor, dystonia, or epilepsy all older than 18 years of age. Whiskers indicate the range of 10 matched analyses, * indicates significant differences among paired comparisons.

We performed two matched analyses using the TriNetX Advance Analytics options to compare relative rates of postsurgical morbidity and mortality (Figures 1B,C, 2, Table 2). The primary analysis compared surgical outcomes between SZ/SAD and PD cohorts matched for the following “presurgical factors” occurring within three months before the index surgery: (a) all 19 CPT surgical procedures; (b) ethnicity; and (c) 39 American College of Surgeons National Surgical Quality Improvement Program (ACS NQIP) presurgical risk factors represented by ICD-10 diagnostic/symptom codes (16). The 39 ACS NQIP codes included: (1) age (<65 and ≥65); 2) gender; (3–11) functional status/ASA class (R40.0-R40.4, R53.2, Z74.09, Z99.1, Z99.8); (12) use of steroids (HS050); (13–15) ascites (R18, K70.11, K70.31); (16–17) smoking (F17.21 and F17.200); (18–19) diabetes (E23.2, HS500); (20) COPD (J44.1); (21) dialysis (R94.4); (22–23) hypertension (I10, I15); (24) disseminated cancer (C80); (25–34) sepsis (R65.2, T81.44, A02.1, A54.86, A41, A40, B37.7, O85, T88.0, T80.211); (35) obesity (E66); and (36–39) congestive heart failure/cardiac disease (I50.2, I50.3, I50.4, Z95.5). Matched analyses varied, and therefore they were repeated 10 times to empirically determine consistency. TrinetX uses a stochastic approach based on propensity matching with logistic regression, and a one-to-one matching with the greedy-nearest-neighbor approach that chooses one group member, then finds the closest match in the other group. Matching for the” presurgical risk factors” above did not occur for 8 factors in some of the runs. The SZ/SAD cohort had 1.09%–1.1% more men in 4/10 runs, 1.21% more non-Hispanics in 2/10 runs, 0.38% more surgical procedures on the respiratory system (1005690) in 1/10 runs, and 0.38%–0.75% more surgical procedures on the nervous system (1009068) in 9/10 runs. The PD cohort had 0.16%–1.21% more essential hypertension (I10), coronary angioplasty implant and graft (Z95.5), surgical procedures on the digestive system (1006964) in 2/10 matching runs each, and 0.50%–1.44% more surgical procedures on the cardiovascular system (1006056) in 7/10 matching runs. Postsurgical outcomes included 61 surgical morbidity factors combined (codes listed in Table 3) and mortality assessed 1 month and 1 year after the index surgery. We used browser-based statistical analysis software, TriNetX Live (TriNetX LLC, Cambridge, MA), for Measures of Association using the *Z*-test result to determine whether the risk for the outcome differed between cohorts.

**Table 2:** Matched analyses of morbidity and mortality data .

(A) PD compared to SZ/SAD						
	Morbidity 1 month*			Mortality 1 month*		
	*n*	%	Range %	*n*	%	Range %
PD	17,914	6.73	6.50–7.00	19,828	2.57	2.50–2.63
SZ/SAD	18,195	9.46	9.33–9.60	19,840	1.55	1.50–1.60
	Morbidity 1 year*			Mortality 1 year*		
	*n*	%	Range %	*n*	%	Range %
PD	17,692	11.13	10.61–11.48	19,176	8.09	7.31–8.33
SZ/SAD	17,972	13.04	12.80–13.27	19,189	3.97	3.56–4.11
**(B) All surgical procedures compared to Neurostimulation**						
	Morbidity 1 month*			Mortality 1 month*		
	*n*	%	Range %	*n*	%	Range %
All	4,060	7.24	5.01–8.16	–	–	–
Stim	4,419	11.06	11.03–11.09	–	–	–
	Morbidity 1 year*			Mortality 1 year*		
	*n*	%	Range %	*n*	%	Range %
All	4,049	13.99	13.31–14.51	4,853	3.08	2.77–3.35
Stim	4,407	15.98	15.89–16.23	4,861	1.21	1.21–1.22

* Significantly different; –, Cases too few to estimate. Averages of 10; Stim = neurostimulator procedures (CPT code 1009276).

**Table 3:** Post-surgical morbidity ICD-10 codes (61) after 1 month and 1 year.

ICD-10 code	Description
D78	Intraoperative and postprocedural complications of the spleen
E36	Intraoperative complications of endocrine system
E89	Postprocedural endocrine and metabolic complications and disorders, not elsewhere classified
G89.18	Other acute postprocedural pain
G97	Intraoperative and postprocedural complications and disorders of nervous system, not elsewhere classified
H21.81	intraoperative Floppy iris syndrome
H52.201	Astigmatism of right eye following operative procedure
H52.202	Astigmatism of left eye following operative procedure
H52.211	Irregular astigmatism of right eye following operative procedure
H52.212	Irregular astigmatism of left eye following operative procedure
H52.213	Bilateral irregular astigmatism; Bilateral irregular astigmatism, postop condition; Irregular astigmatism of bilateral eyes following operative procedure
H59	Intraoperative and postprocedural complications and disorders of eye and adnexa, not elsewhere classified
H95	Intraoperative and postprocedural complications and disorders of ear and mastoid process, not elsewhere classified
I97	Intraoperative and postprocedural complications and disorders of circulatory system, not elsewhere classified
J95	Intraoperative and postprocedural complications and disorders of respiratory system, not elsewhere classified
K91	Intraoperative and postprocedural complications and disorders of digestive system, not elsewhere classified
L76	Intraoperative and postprocedural complications of skin and subcutaneous tissue
M02.0	Arthropathy following intestinal bypass
M61.40	Other calcification of muscle, unspecified site
M61.50	Other ossification of muscle, unspecified site
M96	Intraoperative and postprocedural complications and disorders of musculoskeletal system, not elsewhere classified
N52.33	Erectile dysfunction following urethral surgery
N99	Intraoperative and postprocedural complications and disorders of genitourinary system, not elsewhere classified
O75.4	Other complications of obstetric surgery and procedures
O86.0	Infection of obstetric surgical wound
R50.82	Postoperative fever
T81.31	Disruption of external operation (surgical) wound
T81.32	Disruption of internal operation (surgical) wound
T81.4	Infection following a procedure
T81.9XXA	Unspecified complication of procedure, initial encounter
T81.9XXD	Unspecified complication of procedure, subsequent encounter
T81.9XXS	Unspecified complication of procedure, sequela
T82.6	Infection and inflammatory reaction due to cardiac valve prosthesis
T82.7	Infection and inflammatory reaction due to other cardiac and vascular devices, implants and grafts
T82.8	Other specified complications of cardiac and vascular prosthetic devices, implants and grafts
T82.9	Unspecified complication of cardiac and vascular prosthetic device, implant and graft
T83.5	Infection and inflammatory reaction due to prosthetic device, implant and graft in urinary system
T83.6	Infection and inflammatory reaction due to prosthetic device, implant and graft in genital tract
T83.7	Complications due to implanted mesh and other prosthetic materials
T83.8	Other specified complications of genitourinary prosthetic devices, implants and grafts
T83.9	Unspecified complication of genitourinary prosthetic device, implant and graft
T84.5	Infection and inflammatory reaction due to internal joint prosthesis
T84.6	Infection and inflammatory reaction due to internal fixation device
T84.7	Infection and inflammatory reaction due to other internal orthopedic prosthetic devices, implants and grafts
T84.8	Other specified complications of internal orthopedic prosthetic devices, implants and grafts
T84.9	Unspecified complication of internal orthopedic prosthetic device, implant and graft
T85.7	Infection and inflammatory reaction due to other internal prosthetic devices, implants and grafts
T85.8	Other specified complications of internal prosthetic devices, implants and grafts, not elsewhere classified
T85.9	Unspecified complication of internal prosthetic device, implant and graft
T86	Complications of transplanted organs and tissue
T87	Complications peculiar to reattachment and amputation
T88.8	Other specified complications of surgical and medical care, not elsewhere classified
T88.9	Complication of surgical and medical care, unspecified
Y65.2	Failure in suture or ligature during surgical operation
Y81	General- and plastic-surgery devices associated with adverse incidents
Y82	Other and unspecified medical devices associated with adverse incidents
Y83	Surgical operation and other surgical procedures as the cause of abnormal reaction of the patient, or of later complication, without mention of misadventure at the time of the procedure
Z31.42	Aftercare following sterilization reversal
Z47	Orthopedic aftercare
Z48	Encounter for other postprocedural aftercare
Z91	Personal risk factors, not elsewhere classified
Z91 . 0	Allergy status, other than to drugs and biological substances
Z91.1	Patient's noncompliance with medical treatment and regimen
Z91.4	Personal history of psychological trauma, not elsewhere classified
Z91.5	Personal history of self-harm
Z91.8	Other specified personal risk factors, not elsewhere classified

**Underlined ICD-10 codes **= subset of 12 general/frequent surgical. The surgical morbidity codes were identified by searching ICD-10 codes using asterisks as a wildcard symbol to encompass term variations (terms like *surg*, complication, *operative, *procedure, following, adverse, inciden*, infection, fever, and bypass) (ICD10Data.com).

The second matched analysis compared outcomes between two groups of surgical procedures, neurostimulator procedures including DBS (CPT code 1009276) and all other surgical procedures (Figures 1C, 2C; Table 3). Both surgical cohorts consisted of PD, ET, dystonia, and epilepsy cases. They were matched for diagnoses, and the “presurgical factors” as described above. Of the 61 postsurgical morbidity outcomes considered, only 8 were found at high enough frequencies in the DBS group to allow fair comparison with the “all other surgery” group.

The third matched analysis compared postsurgical DBS morbidity and mortality outcomes among DBS cases with PD, ET, dystonia, and epilepsy (the OCD cohort was too small), matched for 39 presurgical risk factors 1-month and 1-year post-DBS surgical morbidity and mortality outcomes (Figure 1D).

**Figure 3:**
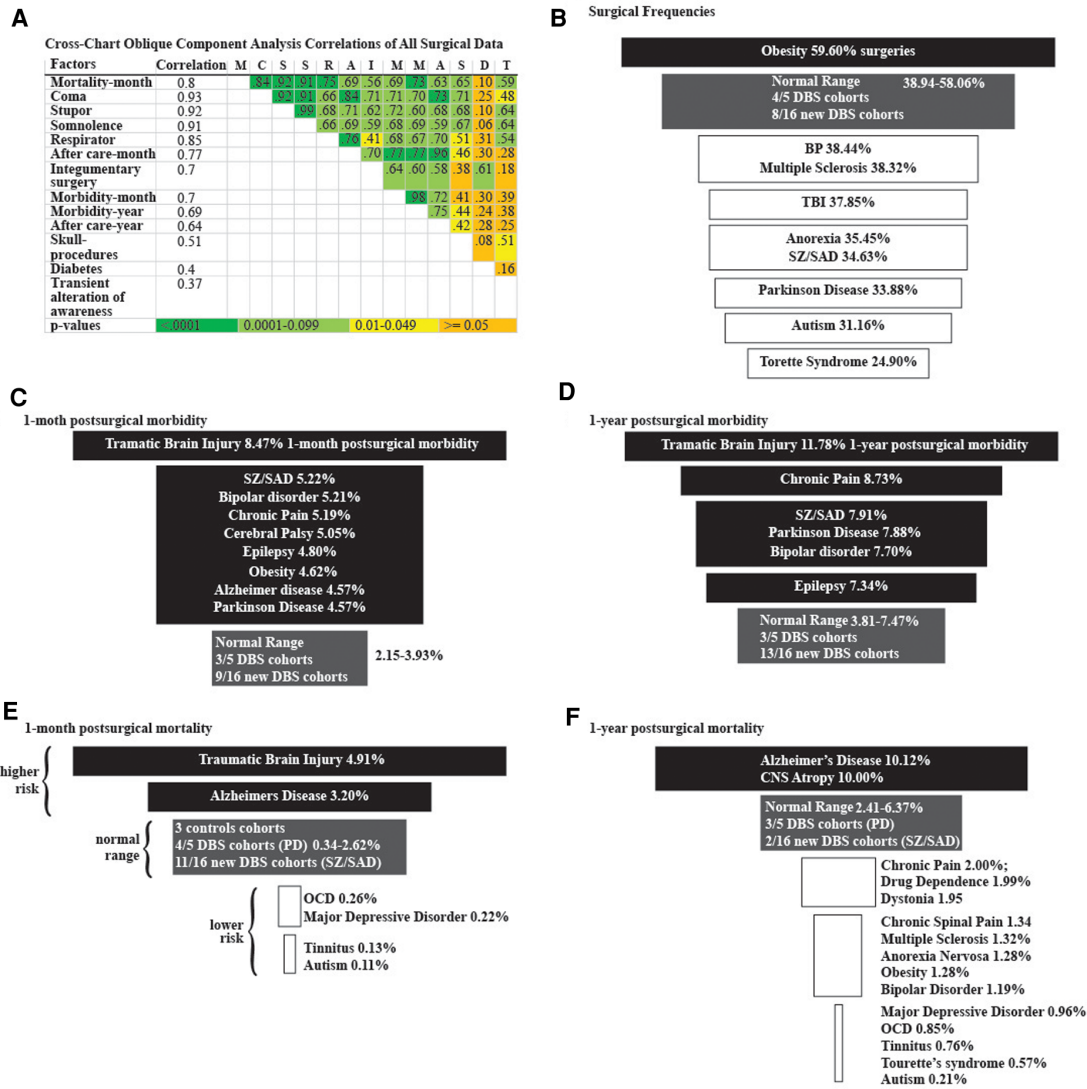
Logistic regression analyses. (**A**) Cross-chart showing the group of 13 highly correlated surgical factors identified from 34,688,986 surgical cases using oblique component analysis. Correlation column is each factor's correlation to the group. Correlations between factors and color-coded *p*-values are listed in the cross-chart. Funnel plots of (**B**) surgical frequencies, **(C**) 1-month postsurgical morbidity, (**D**) 1-year postsurgical morbidity, (**E**) 1-month postsurgical mortality, and (**F**) 1-year postsurgical mortality among diagnostic cohorts. Higher frequencies (black), Normal-range frequencies defined by the results from the three control groups (grey), and lower frequencies (white).

In two final analyses, we used TriNetX to collect frequencies of the “presurgical factors” above and postsurgical outcomes, then used Proc Varclus oblique component analysis (OCA) to determine correlations. We performed a Wilcoxon Rank Sum to determine relative frequencies among the 21 diagnostic and 3 control cohorts (Figures 1E, 3B–F; Tables 3–10; *α *= 0.05; *p* < 0.05; SAS®, Cary, NC). We used this methodology to analyze a total of 73,164,754 (34,688,986 surgical cases) (see Table 4 for the number of surgical cases for each cohort). Only ET, TIN and epilepsy cohorts had equal numbers of women and men. Minorities were more prevalent in six cohorts (SZ/SAD, OB, epilepsy, TIN, autism, and SP) compared to control cohorts (Table 4). Cohorts were unmatched to allow relative comparisons among all cohorts. Within a diagnostic cohort, we excluded all those with comorbid diagnoses considered in this study, except for these 6/21 cohorts where these comorbid conditions were allowed due to low case numbers: anorexia nervosa; autism; TS; OCD; dystonia; and CNS atrophy. We used TriNetX to generate three control cohorts: (1) control cohort 1 (CC1) included all 21 diagnoses explored here, combined to identify outliers among all the brain illnesses considered; (2) control cohort 2 (CC2) consisted of cases who utilized office/outpatient services [code 1013626] to minimize bias towards severe illnesses; and (3) control cohort 3 (CC3) consisted of all ICD-10 diagnoses combined that were made the day before surgery.

**Table 4:** Total surgeries and demographics.

*n*	Female %	White %	Black %	Asian %	Hispanic %
Total 73,164,754	SDC Figure I	SDC Figure II	SDC Figure III	SDC Figure IV	SDC Figure V
CC3 36489450	**AN** **90.31**	**OCD** **85.41**	**SZ** **32.91**	**TIN** **2.99**	**AU** **12.14**
CC2 16026706	**MS** **74.01**	**ET** **85.36**	**OB** **19.06**	**AU** **2.86**	**OB** **12.11**
CC1 12570742	**MDD** **69.15**	**AN** **84.71**	**E** **16.78**	**SP** **2.52**	CC2 10.22
SP 3147987	**AD** **63.39**	**TS** **83.22**	CC1 16.39	PD 2.49	CP 10.00
OB 2152410	**DYS** **63.33**	**PD** **82.53**	CC2 16.08	CP 2.37	CC3 9.51
CHP 581333	**OB** **62.19**	**MDD** **78.20**	CC3 15.00	CC2 2.31	TBI 8.98
TBI 431304	CC2 61.78	**DYS** **77.98**	CP 14.95	CHP 2.18	E 8.30
E 350990	BP 60.98	**CA** **77.88**	AU 14.84	SZ 2.04	DD 8.16
MDD 233742	CC1 60.87	**MS** **77.70**	*SP* *14.54*	DYS 1.96	MDD 7.55
BP 191640	CC3 59.83	**CHP** **75.97**	*BP* *13.15*	AD 1.94	CC1 7.39
TIN 186763	*SP* *59.51*	**AD** **75.85**	*TBI* *12.49*	CC3 1.81	*TIN* *7.03*
DD 125132	*OCD* *57.83*	**TIN** **75.81**	*CHP* *12.46*	MDD 1.65	*CHP* *6.99*
AU 115121	*CP* *56.83*	**DD** **75.52**	*AD* *11.88*	E 1.63	*SP* *6.94*
AD 104324	*ET* *50.82*	**BP** **74.39**	*DD* *11.52*	AN 1.54	*DYS* *5.92*
PD 87532	*TIN* *50.12*	CC1 74.10	*MS* *10.62*	TBI 1.52	*AN* *5.48*
SZ 87209	*E* *49.90*	CC3 73.79	*MDD* *9.99*	CA 1.46	*CA* *5.25*
MS 85510	*CA* *48.57*	CC2 72.30	*DYS* *9.63*	CC1 1.45	*TS* *4.98*
ET 41523	*DD* *47.20*	*SP* *71.91*	*CA* *8.32*	*OB* *1.39*	*SZ* *4.84*
CP 38009	*CP* *45.38*	*TBI* *71.71*	*TIN* *7.65*	*BP* *1.24*	*BP* *4.30*
CA 37195	*TBI* *37.56*	*E* *68.08*	*TS* *7.10*	*ET* *1.23*	*OCD* *3.93*
OCD 32778	*PD* *36.22*	*AU* *67.76*	*OCD* *5.91*	*OCD* *1.14*	*MS* *3.60*
DYS 24732	*SZ* *36.07*	*OB* *67.73*	*PD* *4.85*	*TS* *1.03*	*PD* *2.80*
TS 12352	*TS* *27.96*	*CP* *66.76*	*AN* *4.82*	*MS* *0.65*	*ET* *2.71*
AN 10270	*AU* *20.73*	*SZ* *50.65*	*ET* *4.78*	*DD* *0.51*	*AD* *2.70*

Columns are sorted by descending percentage. AD, Alzheimer's disease; AN, anorexia; AU, autism; BP, bipolar disorder; CP, cerebral palsy; CHP, chronic pain; CC1–3, control cohorts; CA, Huntington's disease CNS atrophy; DD, drug dependence; DYS, dystonia; E, epilepsy; ET, essential tremor; MDD, major depressive disorder; OB, obesity; OCD, obsessive-compulsive disorder; PD, Parkinson's disease; TBI, traumatic brain injury; TIN, tinnitus; MS, multiple sclerosis; SZ, schizophrenia/schizoaffective disorder; SP, chronic spinal pain; TS, Tourette's syndrome; black outline = cohorts statistically indistinct from control groups, **bold **= cohorts with a higher frequency than controls, *italics *= cohorts with lower frequency than controls, grey outline = cohorts with equal numbers of men and women.

## Results

The percentage of patients who died after a surgical procedure (postsurgical mortality) was lower in the SZ/SAD cohorts compared to the matched PD cohorts in all 10 matched analyses at 1 month (average difference, −1.02%) and 1 year (average difference, −4.11%) after surgery (Figure 2A; Table 2A). The frequency of the 61 postsurgical morbidity events combined was higher in the SZ/SAD cohorts in all 10 matched analyses at both 1 month (average difference +2.73%) and 1 year (average difference +1.91%) after surgery (Figures 1A,B, 2A; Table 2A). The SZ/SAD cohort had a 4% higher frequency of personal risk factors (Z91) post-surgically, including noncompliance with medical treatment regimen (Z91.1) at both 1 month and 1 year after surgery (Table 2A). The number of instances of more than one postsurgical morbidity event per person was 0.033–0.086% higher after 1 month in all 10 runs and 0.022–0.029% higher in 5/10 runs in the SZ/SAD cohort compared to the matched PD cohort. The most common surgical procedures were cardiovascular procedures (56%) and <2% were brain surgeries (Figure 2B).

For the neurostimulator procedures (CPT 1009276) morbidity/mortality matched comparison to all other surgeries (CPT 1003143), mortality was 1.87% lower in the neurostimulation cohort after 1 year compared to the “all surgeries except DBS” cohort (Figures 1C, 2C; Table 2B). The frequency of postsurgical morbidity events combined was higher for DBS surgeries at 1 month (+3.82%) and 1 year (+2.00%) than all other surgeries combined in PD, ET, DYS, and epilepsy cases matched for “presurgical factors” (Figures 1C, 2C; Table 2B). We selected this PD, ET, DYS, and epilepsy cohort because this group is often treated with DBS. The higher postsurgical morbidity included the (Y83) code “surgical operation and other surgical procedures as the cause of abnormal reaction of the patient, or of later complication, without mention of the misadventure at the time of the procedure” and the specifier, “implant of an artificial internal device” (Y83.1) code (Figure 2A). Among matched DBS cohorts, the 1-month and 1-year post-DBS morbidity (11.4%–23.6%) and mortality rates (0%–4.9%) were similar among PD (*n* = 550–770), epilepsy (*n* = 666), ET (*n* = 755) and dystonia (*n* = 489) cohorts that had DBS surgery.

In the 21 diagnostic and 3 control cohorts, we examined frequencies of all demographics, risk factors, surgeries and outcomes and identified 13 correlated factors, including 6/39 presurgical risk factors, 2/19 surgery types, and 2/59 postsurgical morbidity outcomes and mortality at a month (Figure 3A). The frequencies of 5 factors out of 122 were highly correlated (*r *= 0.37–0.93) with each other (1-month postsurgical mortality, 1-year postsurgical morbidity, presurgical coma, stupor somnolence, and being on a respirator) (Figure 3A).

We identified disparities in the frequency of all surgical procedures combined (ICD code 1003143) in the TS, autism, PD, SZ/SAD, anorexia nervosa, TBI, MS, and BP cohorts (24.90%–38.44%) compared to control cohorts (38.94%–58.06%) (Figure 3B; Table 5, surgery %). Subjects with SZ/SAD received 16.1% fewer surgeries than the average of the control cohorts and were in the lowest surgical frequency group for all surgery subtypes except cardiovascular and digestive surgeries. Of the 39 presurgical risk factors considered, 13 were common enough for further analysis (Table 5). We found higher levels of presurgical risk factors in AD (6/13), TBI (4/13), epilepsy (4/13), and SZ/SAD (2/13). The SZ/SAD cohort had a relatively high frequency of nicotine dependence (8.40%) and being in a stupor (0.71%) compared to controls. Control cohorts were among the cohorts with the highest frequency of presurgical risk factors (5/13), including dyspnea, systolic heart failure, machine dependence, ascites, and secondary hypertension.

**Table 5:** Logistic regression analysis of all surgical frequencies and presurgical risks.

**Surgery**	**13/39 Common Presurgical Risks %**												
**%**	**≥65 yrs.**	**1° HT**	**Nicot**	**Dysp**	**Coma**	**Sepsis**	**Somn**	**Stupor**	**Heart**	**Mach**	**Ascites**	**Aware**	**2° HT**
Supplementary Digital Content (SDC) Figures													
VI	VII	VIII	IX	X	XI	XII	XIII	XIV	XV	XVI	XVII	XVIIII	XIX
**OB** **59.60**	**AD** **95.59**	**AD** **48.21**	**DD 16.09**	CC3 8.97	**TBI** **7.44**	**AD** **5.93**	**TBI** **2.50**	**TBI** **2.35**	AD 1.93	CC1 1.80	CC3 1.61	**E** **1.15**	CC1 .35
CC1 58.06	**PD** **83.03**	CC1 37.71	**BP** **9.06**	CA 7.93	**E** **1.70**	CC3 3.43	**AD** **1.13**	**AD** **1.01**	CC3 1.88	CC3 ­­­1.53	DD .98	**TBI** **.61**	CC3 .31
CC2 56.74	**ET** **57.20**	PD 36.22	**SZ** **8.40**	CC1 7.53	CC3 1.20	PD 3.24	**E** **.97**	**E** **.89**	CC1 1.44	OB 1.50	CC1 .90	**AU** **.60**	CC2 .29
CHP 55.97	**CA** **41.06**	ET 33.25	CC3 5.34	CC2 6.13	CC1 .96	DD 3.11	SZ .83	**SZ** **.71**	PD 1.30	CA 1.37	AN .89	**AD** **.54**	*OB* *.21*
TIN 5.67	**DYS** **32.63**	CC3 28.01	CC1 4.57	*OB* *4.64*	AD .89	E 2.86	CC1 .74	DD .54	OB 1.14	AD 1.19	SZ .72	TS .51	*AD* *.21*
DYS 49.17	**TIN** **31.21**	CC2 27.26	CHP 4.56	*ET* *4.29*	SZ .69	SZ 2.60	DD .72	CC1 .53	CC2 .94	ET 1.07	CHP .63	SZ .40	TS .20
SP 48.66	CC1 29.05	*CA* *24.61*	E 4.04	*AD* *3.95*	PD .64	CC1 2.27	CC3 .62	CA .53	*E* *.88*	PD 1.02	CC2 .60	DYS .39	*E* *.18*
ET 45.80	CC3 28.01	*CHP* *23.57*	TBI 3.53	*PD* *3.84*	DD .61	CA 2.01	PD .61	PD .51	SZ .82	CHP 1.01	E .57	CC3 .37	*CA* *.18*
CP 44.23	CHP 26.69	*SZ* *22.21*	MDD 3.05	*DD* *3.82*	CA .46	CP 1.74	CA .60	CC3 .49	ET .79	DYS .77	*OB* *.50*	PD .29	AN .18
MDD 43.37	CC2 25.82	*TIN* *21.42*	OCD 3.03	*SZ* *3.50*	BP .38	OB 1.42	DYS .55	BP .38	*DD* *.78*	E .72	*TBI* *.47*	CC1 .29	*DD* *.16*
AD 41.88	*SP* *23.40*	*DYS* *18.19*	OB 2.81	*TIN* *3.33*	DYS .34	CP 1.37	BP .47	DYS .35	*CHP* *.73*	CC2 .67	*BP* *.45*	CP .28	*ET* *.16*
OCD 4.83	*TBI* *18.30*	*BP* *17.68*	SP 2.55	*DYS* *3.26*	OCD .27	BP 1.16	MDD .39	TS .23	*CA* *.68*	*OCD* *.58*	*ET* *.39*	CA .27	*DYS* *.16*
CA 4.73	*OB* *16.27*	*E* *17.63*	MS 2.17	*SP* *3.20*	ET .26	MS 1.10	TS .37	ET .22	*TBI* *.46*	*SZ* *.54*	*AD* *.35*	OCD .24	*CHP* *.15*
DD 4.56	*E* *15.77*	*MDD* *17.62*	TS 2.09	*CHP* *3.11*	TS .23	ET 1.06	AN .35	MDD .22	*BP* *.41*	*MDD* *.51*	*MDD* *.34*	AN .21	*MDD* *.13*
E 39.55	*MDD* *14.93*	*OB* *14.54*	DYS 2.07	*E* *2.99*	OB .23	DYS 1.00	OCD .34	AN .19	*MDD* *.40*	*BP* *.47*	*PD* *.33*	DD .21	*TBI* *.13*
CC3 38.94	*SZ* *14.53*	*OCD* *14.54*	AN 1.94	*OCD* *2.91*	CHP .23	TBI .79	ET .30	OCD .19	*DYS* *.38*	*CP* *.43*	*CA* *.33*	BP .19	*CP* *.13*
*BP* *38.44*	*MS* *14.43*	*TBI* *13.77*	CA 1.88	*BP* *2.77*	MDD.22	AN .79	CC2 .26	SP .18	*AN* *.35*	*DD* *.42*	*SP* *.27*	ET .13	*OCD* *.12*
*MS* *38.32*	*OCD* *8.89*	*MS* *12.99*	CC2 1.52	*MDD* *2.56*	AN .19	OCD .71	*OB* *.24*	MS .16	*OCD* *.32*	*MS* *.36*	*DYS* *.24*	MDD .12	*PD* *.11*
*TBI* *37.85*	*BP* *8.68*	*DD* *11.12*	ET 1.51	*AN* *2.56*	SP .19	CC2 .57	*MS* *.24*	CC2 .14	*SP* *.32*	*TIN* *.35*	*CP* *.21*	MS .10	*TIN* *.11*
*AN* *35.45*	*CP* *5.02*	*TS* *8.06*	*TIN* *1.19*	*TBI* *2.54*	MS .18	*MDD* *.49*	*SP* *.23*	*CHP* *.12*	*TIN* *.24*	*TBI* *.32*	*TS* *.20*	CC2 .092	*BP* *.11*
*SZ* *34.63*	*DD* *3.91*	*CP* *8.04*	*PD* *1.03*	*CP* *2.32*	CP .17	*SP* *.46*	*CHP* *.21*	*OB* *.10*	*MS* *.23*	*SP* *.29*	*OCD* *.19*	*CHP* *.077*	*SZ* *.10*
*PD* *33.88*	*TS* *3.88*	*AN* *4.48*	*AD* *.97*	*TS* *1.93*	AU .17	*TS* *.33*	*CP* *.15*	CP .095	*TS* *.20*	*AN* *.25*	*MS* *.18*	*SP* *.058*	*SP* *.10*
*AU* *31.16*	*AN* *3.1*	*SP* *2.20*	*CP* *.57*	*MS* *1.76*	CC2.096	*AU* *.29*	*TIN* *.15*	*TIN* *.052*	*CP* *.15*	*TS* *.23*	*TIN* *.12*	*OB* *.039*	*MS* *.090*
*TS* *24.90*	*AU* *.39*	*AU* *1.50*	*AU* *.22*	*AU* *1.28*	*TIN* *.069*	*TIN* *.19*	*AU* *.08*	*AU* *.042*	*AU* *.063*	*AU* *.10*	*AU* *.09*	*TIN* *.037*	*AU* *.071*

Sepsis = A41 Other sepsis; Nicot = Nicotine dependence; 1° HT = I10 Essential hypertension; Dysp = R06.0 Dyspnea; Heart = I5.2 Systolic heart failure; 2 HT°= I15 secondary hypertension; Ascites = R18 Ascites; Somn = R4.0 somnolence; Stupor =  R4.1 Stupor; Coma = R4.2 Coma; Aware = R4.4 transient alteration of awareness; Mach = Z99.8 Dependence on machine/device. Bold boxes = cohorts that are statistically indistinct from or within the range of the three control cohorts. Bold = statistically higher than the 3 control groups. *Italics *= statistically lower than the 3 control cohorts CC1-3. The 39 ACS NQIP codes are included in the methods section. Statistically indistinct frequencies indicated with shared colors in adjacent cells. Columns are sorted by descending percentage. AD, Alzheimer's disease; AN, anorexia; AU, autism; BP, bipolar disorder; CA, Huntington's disease and CNS atrophy; CP, cerebral palsy; CHP, chronic pain; CC1–3, control cohorts; DD, substance dependence; DYS, dystonia; E, epilepsy; ET, essential tremor; MDD, major depressive disorder; OB, obesity; OCD, obsessive-compulsive disorder; PD, Parkinson's disease; TBI, traumatic brain injury; TIN, tinnitus; MS, multiple sclerosis; SZ, schizophrenia and schizoaffective disorder; SP, chronic spinal pain; TS, Tourette's syndrome.

Surgical morbidity events in combination were higher in the TBI (8.5%), and the SZ/SAD, BP, CHP, and CP cohorts (5.1%–5.2%), compared to the epilepsy, OB, AD, and PD cohorts (4.6%–4.8%) and the three control cohorts (1.43%–3.79%) after one month (Figures 3C, Table 7). After 1 year, morbidity was higher in the TBI (11.78%) and CHP (8.73%) cohorts than the SZ/SAD, PD, BP, and CP cohorts (7.47%–7.90%) and the three control cohorts (3.81%–7.04%) (Figure 3D; Tables 6, 8). In the obesity and TBI cohorts, morbidity was higher in 4/8 of the more common surgical morbidity events examined individually (Table 6). Control cohorts had lower frequencies in 3/8 of the common surgical morbidity outcomes examined individually. Much of the increased frequency of postsurgical morbidity in the SZ/SAD cohort was accounted for by the code Z91.1 (postsurgical noncompliance with medical treatment and regimen); This event occurred more frequently in SZ/SAD, epilepsy, BP, substance dependence, MDD, obesity, AD, and PD (0.27%–3.17%) compared to control groups (0.03%–0.26%).

**Table 6:** Postsurgical morbidity.

**Postsurgical Morbidity (Mb) %**																	
Combined		Eight common postsurgical morbidity events individually															
Mb mo.	Mb yr.	Pain mo.	Pain yr.	Skin mo.	Skin yr.	Wnd mo.	Wnd yr.	Inf mo.	Inf yr.	Comp mo.	Comp yr.	Care mo.	Care yr.	Pers mo.	Pers yr.	Circ mo.	Circ yr.
XX	XXI	XXII	XXIII	XXIV	XXV	XXVI	XXVII	XXVIII	XXIX	XXX	XXXI	XXXII	XXXIII	XXXIV	XXXV	XXXVI	XXXVII
**TBI** **8.47**	**TBI** **11.78**	**CHP** **1.37**	**CHP** **2.07**	TS .20^	TS .20^	CP .079^	**E** **.85**	CP .079	TS .20^	**PD** **.38**	**PD** **.59**	**TBI** **3.17**	**TBI** **4.27**	**SZ** **3.10**	**SZ** **4.74**	**TS** **.17**^	CC3 .20
**SZ** **5.22**	**CHP** **8.73**	**CP** **.87**	**CP** **1.09**	AN .18^	CC3 .18	ET .070^	TS .20^	ET .070^	AN .18^	**CP** **.33**	**E** **.48**	CC3 1.86	CC3 3.18	**BP** **2.59**	**AD** **4.02**	**AN** **.15**^	TBI .20
**BP** **5.21**	**SZ** **7.91**	**OB** **.73**	**OB** **.95**	OB .11	AN .18^	**OB** **.070**	**OB** **.13**	**OB** **.070**	**OB** **.14**	**E** **.32**	**CP** **.45**	OB 1.39	OB 2.29	**AD** **2.49**	**BP** **3.81**	**TBI** **.15**	E .19
**CHP** **5.19**	**PD** **7.88**	**TBI** **.70**	**TBI** **.90**	CC3 .10	OB .16	BP .060	CP .10	BP .060	BP .12	**OB** **.31**	**OB** **.43**	CP 1.24	CC1 2.036	**E** **2.08**	**PD** **3.31**	**E** **.14**	TS .17^
**CP** **5.05**	**BP** **7.70**	DD .57	**DD** **.85**	TBI .10	TBI .13	E .048	CC1 .10	E .050	TBI .11	**CHP** **.30**	**CHP** **.42**	DD 1.23	CP 2.013	**PD** **1.78**	**E** **3.23**	CC3 .11	OB .16
**E** **4.80**	CP 7.47	CC3 .51	CC3 .76	CP .095^	E .12	CA .047^	CC3 .099	CA .048	CC3 .095	**BP** **.25**	**AD** **.37**	AU 1.13	CHP 2.00	**MDD** **1.52**	**MDD** **2.61**	OB .11	ET .16
**OB** **4.62**	**E** **7.34**	TS .51	CC1 .74	SZ .089	CC1 .12	SZ .044	CHP .096	SZ .047	CC1 .090	**TS** **.23**	**BP** **.34**	BP 1.11	AU 1.79	**DD** **1.39**	**DD** **2.47**	CP .11	AN .15^
**AD** **4.57**	AD 7.21	BP .49	BP .65	E .079	PD .12	CC3 .042	BP .081	CC3 .043	DD .085	**AD** **.23**	**CA** **.34**	E 1.08	DD 1.77	**TBI** **1.24**	**OCD** **2.29**	CA .099	PD .15
**PD** **4.57**	OB 7.17	E .43	CHP .63	BP .077	SZ .12	CC1 .042	PD .078	CC1 .042	CHP .081	**SZ** **.21**	TS .29	TS 1.073	PD 1.77	**OCD** **1.18**	**TBI** **2.08**	PD .098	CA .15
DD 3.93	CC3 7.04	CC1 .42	TS .62	DD .072^	DD .11	AU .041^	TBI .076	AU .042	CP .078	**CA** **.20**	**TBI** **.27**	CHP 1.07	E 1.74	**OB** **1.15**	**CA** **2.06**	SZ .097	AD .14
CC3 3.79	DD 6.29	CS .39	E .62	ET .070^	CP .11	PD .040^	ET .070^	PD .041	E .077	AN .18^	**SZ** **.26**	CC1 1.01	TS 1.72	**CHP** **1.15**	**AU** **2.02**	ET .091	CP .14
AU 3.36	CC1 5.93	AU .38	AU .54	MS .063^	BP .11	CHP .03	SZ .064	CHP .040	ET .070	**ET** **.17**	ET .25	PD 1.00	BP 1.68	**AU** **1.07**	**CHP** **2.01**	AD .086	SZ .13
TS 3.24	CA 5.85	CA .36	CC2 .51	CA .062^	MS .10	TBI .035	DD .061	TBI .039	SZ .068	**TBI** **.16**	DD .23	SZ 1.00	SP 1.66	**CP** **.94**	AN 1.88	AU .081	BP .11
MDD 3.00	AU 5.45	AN .28	CA .49	PD .062^	ET .10^	MS .033^	SP .058	MS .035	PD .062	**MS** **.16**	DYS .21	SP .94	TIN 1.65	**CA** **.88**	OB 1.82	BP .072	DD .10
CA 2.96	MDD 5.31	SZ .27	AN .46	CC1 .061	CHP .092	DYS .033^	CA .053	DYS .033^	MS .060	**DD** **.14**	MS .20	TIN .92	SZ 1.46	**AN** **.82**	TS 1.54	DD .065	CC1 .093
OCD 2.86	TS 5.29	PD .27	*MDD* *.42*	AU .055^	CA .091	OCD .032^	AU .050	OCD .033	SP .053	AU .13	SP .20	MDD .79	CC2 1.42	**MS** **.74**	CP 1.46	CC1 .052	AU .088
SP 2.83	SP 5.19	MDD .27	*DYS* *.41*	CHP .049	SP .088	AD .028^	CC2 .050	AD .032	CA .053	**SP** **.11**	CC1 .19	OCD .77	OCD 1.42	**ET** **.66**	DYS 1.34	TIN .052	TIN .087
CC1 2.73	OCD 5.19	AD .25	*PD* *.39*	SP .049	AD .081	DD .026^	MDD .047	DD .028	AD .048	OCD .11	AN .19	AD .68	MDD 1.39	TS .55	ET 1.30	MS .05	MS .087
MS 2.73	ET 4.91	CC2 .25	*TIN* *.37*	AD .039^	CC2 .069	SP .021	OCD .042	SP .026	MDD .044	CC1 .10	AU .17	CC2 .64	*DYS* *1.088*	**DYS** **.53**	CC1 1.29	SP .048	SP .085
ET 2.66	AN 4.41	TIN .23	*SZ* *.35*	DYS .036^	MDD .062	CC2 .016	MS .042	AN .18^	AU .042	TIN .093	OCD .17	MS .60	*AD* *1.09*	**SP** **.50**	MS 1.24	CHP .046	CHP .082
TIN 2.16	MS 4.21	MS .21	*AD* *.33*	TIN .035^	TIN .060	TIN .015^	AD .042	TS .20^	TIN .039	DYS .086	CC3 .16	*DYS* *.54*	*MS* *1.01*	**TIN** **.45**	SP 1.07	DYS .032	MDD .066
AN 2.15	TIN 3.97	DYS .21	*ET* *.31*	OCD .033^	AU .059	MDD .015^	DYS .033^	CC2 .016	OCD .036	CC3 .073	TIN .14	AN .46	*AN* *.98*	CC1 .38	CC3 1.04	MDD .03	CC2 .064
DYS 1.75	DYS 3.93	ET .20	*MS* *.30*	MDD .026^	DYS .053^	AN 0	TIN .032	TIN .015	DYS .033	MDD .051	MDD .10	*CA* *.20*	*CA* *.34*	CC3 .33	TIN .94	OCD .028^	*DYS* *.054*
CC2 1.43	CC2 3.81	*OCD* *.03^*	*OCD* *.05*	CC2 .025	OCD .049	TS 0	AN 0	MDD .015	CC2 .032	CC2 .029	CC2 .073	*ET* *.17*	*ET* *.25*	CC2 .18	CC2 .91	CC2 .024	*OCD* *.053*

Mt = Mortality; Mb = Morbidity; Pain = G89.18 Other acute postprocedural pain; Skin = L76 Intraoperative and postprocedural complications of skin and subcutaneous tissue; Wnd = T81.31 Disruption of external operation surgical wound, not elsewhere classified; Inf = T81.4 Infection following a procedure; Comp = Y83 Surgical operation and other surgical procedures as the cause of abnormal reaction of the patient, or of later complication, without mention of misadventure at the time of the procedure; Care = Z48 Encounter for other postprocedural aftercare; Pers = Z91 Personal risk factors, not elsewhere classified; Circ = I97 Intraoperative and postprocedural complications and disorders of circulatory system, not elsewhere classified; mo. = 1 month after surgery; yr.= 1 year after surgery; **Bold **= statistically higher than the 3 control groups. *Italics *= statistically lower than the 3 control cohorts CC1-3. ^ = data 10 or less (TrinetX limitation). Statistically indistinct frequencies indicated with shared colors in adjacent cells. Columns are sorted by descending percentage. AD, Alzheimer's disease; AN, anorexia; AU, autism; BP, bipolar disorder; CA, Huntington's disease and CNS atrophy; CP, cerebral palsy; CHP, chronic pain; CC1–3, control cohorts; DD, substance dependence; DYS, dystonia; E, epilepsy; ET, essential tremor; MDD, major depressive disorder; OB, obesity; OCD, obsessive-compulsive disorder; PD, Parkinson's disease; TBI, traumatic brain injury; TIN, tinnitus; MS, multiple sclerosis; SZ, schizophrenia and schizoaffective disorder; SP, chronic spinal pain; TS, Tourette's syndrome.

**Table 7:** Morbidity 1 month postsurgical. Statistically indistinct frequencies indicated with shared colors in adjacent cells. Columns are sorted by descending percentage.

	Total	Obs %	TBI	SZ	BP	CHP	CP	E	OB	AD	PD	DD	CC3	AU	TS	MDD	CA	OCD	SP	CC1	MS	ET	TIN	AN	DYS	CC2
TBI	110937	8.5																								
SZ	23542	5.2																								
BP	65864	5.2																								
CHP	211895	5.2																								
CP	12682	5.1																								
E	107758	4.8																								
OB	939595	4.6																								
AD	31030	4.6																								
PD	24224	4.6																								
DD	36205	3.9																								
CC3	864922	3.8																								
AU	23821	3.4																								
TS	5126	3.2																								
MDD	65000	3.0																								
CA	20936	3.0																								
OCD	30594	2.9																								
SP	1062244	2.8																								
CC1	1121847	2.7																								
MS	27800	2.7																								
ET	14338	2.7																								
TIN	64839	2.2																								
AN	5709	2.2																								
DYS	29269	1.7																								
CC2	1065782	1.4																								

AD, Alzheimer's disease; AN, anorexia; AU, autism; BP, bipolar disorder; CA, Huntington's disease and CNS atrophy; CP, cerebral palsy; CHP, chronic pain; CC1–3, control cohorts; DD, substance dependence; DYS, dystonia; E, epilepsy; ET, essential tremor; MDD, major depressive disorder; OB, obesity; OCD, obsessive-compulsive disorder; PD, Parkinson's disease; TBI, traumatic brain injury; TIN, tinnitus; MS, multiple sclerosis; SZ, schizophrenia and schizoaffective disorder; SP, chronic spinal pain; TS, Tourette's syndrome.

**Table 8:** Morbidity 1 year postsurgical. Logistic regression analysis of frequencies of postsurgical mortality from Figure 3F; Statistically indistinct frequencies indicated with shared colors in adjacent cells. Columns are sorted by descending percentage.

	Total	Obs %	TBI	CHP	SZ	PD	BP	CP	E	AD	OB	CC3	DD	CC1	CA	AU	MDD	TS	SP	OCD	ET	AN	MS	TIN	DYS	CC2
TBI	110937	11.78																								
CHP	211895	8.73																								
SZ	23542	7.90																								
PD	24224	7.88																								
BP	65724	7.70																								
CP	12682	7.47																								
E	107758	7.34																								
AD	31030	7.21																								
OB	939568	7.17																								
CC3	864922	7.04																								
DD	36205	6.29																								
CC1	1121847	5.93																								
CA	20936	5.85																								
AU	23821	5.45																								
MDD	64773	5.31																								
TS	5126	5.29																								
SP	1062244	5.19																								
OCD	30594	5.19																								
ET	14338	4.91																								
AN	5709	4.41																								
MS	27806	4.22																								
TIN	64839	3.97																								
DYS	29269	3.93																								
CC2	1065782	3.81																								

AD, Alzheimer's disease; AN, anorexia; AU, autism; BP, bipolar disorder; CA, Huntington's disease and CNS atrophy; CP, cerebral palsy; CHP, chronic pain; CC1–3, control cohorts; DD, substance dependence; DYS, dystonia; E, epilepsy; ET, essential tremor; MDD, major depressive disorder; OB, obesity; OCD, obsessive-compulsive disorder; PD, Parkinson's disease; TBI, traumatic brain injury; TIN, tinnitus; MS, multiple sclerosis; SZ, schizophrenia and schizoaffective disorder; SP, chronic spinal pain; TS, Tourette's syndrome.

When considering all 21 diagnostic and 3 control cohorts in the unmatched analysis, mortality was highest in the TBI (4.91%) and AD (3.20%) cohorts one month after surgery (Figure 3E; Table 9). At one year after surgery, the AD (10.12%) and CNS atrophy (10.00%) cohorts had the highest mortality (Figure 3F; Table 10). Mortality was lowest in OCD, MDD, tinnitus, and autism (0.10%–0.34%) after one month. At 1 year, mortality was also low in CP, substance dependence, chronic spinal pain, CP, MS, autism, obesity, BP, and TS (0.21%–1.99%). Frequencies of surgical mortality in the remaining cohorts (0.41%–1.90% at 1 month and 2.96%–6.24% at 1 year) were not statistically different from or within the range of the three control cohorts (0.35%–2.62% at a month and 2.60%–3.67% at a year) and the PD cohort (1.76% at 1 month and 6.24% at 1 year) (Tables 9, 10). For the SZ/SAD cohort, postsurgical mortality (1.32%–2.97%) was lower than the PD and 1–2 control cohorts but was higher than 15 other diagnostic cohorts (0.11%–2.41%).

**Table 9:** Mortality 1 month postsurgical. Statistically indistinct frequencies indicated with shared colors in adjacent cells. Columns are sorted by descending percentage.

	Total	Obs %	TBI	AZ	CC3	CNS	E	PD	SZ	CC1	DD	ET	CHP	OB	AN	CP	BP	MS	DYS	CC2	SP	OCD	TS	MDD	TIN	AU
TBI	110937	4.91																								
AD	31030	3.20																								
CC3	864922	2.62																								
CNS	20936	1.90																							
E	107758	1.82																								
PD	24224	1.76																								
SZ	23542	1.32																								
CC1	1121847	1.24																								
DD	36205	0.67																								
ET	14338	0.64																								
CHP	211895	0.59																								
OB	939568	0.52																								
AN	5709	0.49																								
CP	12682	0.48																								
BP	65724	0.44																								
MS	27806	0.44																								
DYS	29269	0.41																								
CC2	1065782	0.35																								
SP	1062244	0.34																								
OCD	30594	0.26																								
TS	5126	0.22																							
MDD	64773	0.21																								
TIN	64839	0.13																								
AU	23821	0.11																								

AD, Alzheimer's disease; AN, anorexia; AU, autism; BP, bipolar disorder; CA, Huntington's disease and CNS atrophy; CP, cerebral palsy; CHP, chronic pain; CC1–3, control cohorts; DD, substance dependence; DYS, dystonia; E, epilepsy; ET, essential tremor; MDD, major depressive disorder; OB, obesity; OCD, obsessive-compulsive disorder; PD, Parkinson's disease; TBI, traumatic brain injury; TIN, tinnitus; MS, multiple sclerosis; SZ, schizophrenia and schizoaffective disorder; SP, chronic spinal pain; TS, Tourette's syndrome.

**Table 10:** Mortality 1 year postsurgical. Statistically indistinct frequencies indicated with shared colors in adjacent cells. Columns are sorted by descending percentage.

	Total	Obs %	AD	CNS	CC3	PD	TBI	E	CC1	SZ	CC2	ET	CHP	DD	DYS	SP	CP	MS	AN	OB	BP	MDD	OCD	TIN	TS	AU
AD	31030	10.12																								
CNS	20936	10.00																								
CC3	864922	6.37																								
PD	24224	6.24																								
TBI	110937	6.23																								
E	107758	3.95																								
CC1	1121847	3.64																								
SZ	23542	2.97																								
CC2	1065782	2.60																								
ET	14338	2.41																								
_CHP_	211895	2.00																						
DD	36205	1.99																							
DYS	29269	1.95																							
SP	1062244	1.40																								
CP	12682	1.34																								
MS	27806	1.32																								
AN	5709	1.28																								
OB	939568	1.28																								
BP	65724	1.19																								
MDD	64773	0.96																								
OCD	30594	0.85																								
TIN	64839	0.76																								
TS	5126	0.57																								
AU	23821	0.21																								

AD, Alzheimer's disease; AN, anorexia; AU, autism; BP, bipolar disorder; CA, Huntington's disease and CNS atrophy; CP, cerebral palsy; CHP, chronic pain; CC1–3, control cohorts; DD, substance dependence; DYS, dystonia; E, epilepsy; ET, essential tremor; MDD, major depressive disorder; OB, obesity; OCD, obsessive-compulsive disorder; PD, Parkinson's disease; TBI, traumatic brain injury; TIN, tinnitus; MS, multiple sclerosis; SZ, schizophrenia and schizoaffective disorder; SP, chronic spinal pain; TS, Tourette's syndrome.

## Discussion

Advanced therapeutics like DBS have the potential to significantly improve symptoms and quality of life in those with treatment-refractory SZ/SAD, as has been shown for PD, ET, dystonia, epilepsy, and OCD. While early studies have been promising, more clinical trials are essential to assess the effectiveness of DBS for treatment-refractory SZ/SAD, and we endeavored to elucidate diagnosis-related surgical risk to address ethical concerns surrounding DBS clinical trials. Here we showed that with or without extensive matching for presurgical risk factors, subjects in the SZ/SAD cohort had lower postsurgical mortality compared to the matched PD cohort after 1 month and 1 year. In addition, the PD and SZ/SAD cohorts had higher postsurgical mortality than most of the other diagnostic cohorts even though they both were within the range of the control cohorts. Both SZ/SAD and PD have been shown in independent studies to have higher frequencies of adverse surgical outcomes relative to control populations without these conditions (31–49). Weighting of disability of schizophrenia (weight 0.399–0.894) and PD (weight 0.011–0.711) overlap (55). Postsurgical mortality was within the range of the control groups or lower in all 21 diagnostic cohorts except TBI and AD after 1 month, and AD and central nervous system atrophy after 1 year. Therefore, these postsurgical mortality findings are favorable results for most new DBS indications examined.

In the matched analysis of postsurgical morbidity, two of the most relevant adverse events for DBS surgery, infection, and hemorrhage, were not more frequent in the SZ/SAD cohort compared to the PD cohort. Postsurgical morbidity was higher in the SZ/SAD cohort than the PD cohort after 1 month, with or without matching for presurgical factors but not after 1 year. Postsurgical morbidity for most (12–16) of the 21 diagnostic cohorts was within the range of the control cohorts but higher in TBI, SZ/SAD, BP, chronic pain, cerebral palsy, epilepsy, obesity, AD and PD cohorts. Consistent with other investigations showing lower medication adherence in subjects with schizophrenia or depression, non-compliance with the medical treatment and regimen (Z91.1) occurring within the postsurgical window (counted as a morbidity event in this analysis) was more frequent in the SZ/SAD cohort (56, 57). To improve outcomes, providers and researchers need to identify the reasons for non-adherence and develop interventions that minimize barriers to engagement in care. Though overall morbidity and mortality were correlated with each other, the increased postoperative morbidity in the SZ/SAD cohort did not translate to higher mortality relative to the PD and control cohorts.

Surgical inequities were identified among mental illness diagnoses (BP, SZ/SAD, anorexia nervosa, autism, and Tourette's syndrome), as well as neurological conditions (MS and PD) and trauma (TBI). Lower surgical frequency may reflect higher severity in the TBI cohort; this cohort had the highest presurgical coma, postsurgical morbidity, and mortality (1 month after surgery). Lower surgical frequency in the BP and SZ/SAD cohorts may have been related, in part, to high presurgical nicotine dependence and surgeons refusing to operate on smokers, as it is known to increase adverse outcomes across surgical specialties (58–60). In addition, 40% of subjects with SZ/SAD have negative symptoms that make it difficult for them to seek out medical care, including surgical interventions, without assistance (61). It is unclear why surgical disparities exist for the anorexia nervosa, autism, Tourette's syndrome and MS cohorts; their adverse postsurgical outcomes were within or below the control range. Surgical disparities reflect inequalities in the application of the ethical principal of justice and may be related to inaccessibility, healthcare disparities, higher perceived surgical risks, concerns over capacity to consent to surgical procedures (encompassing understanding, appreciating, reasoning, and evidencing a choice), and paternalistic overprotection of vulnerable subjects (11–14, 62–67). It is important to identify and rectify, when possible, unjust disparities in access to surgical procedures in populations with refractory illness who experience significant suffering, disability, and poor quality of life.

These diagnosis-specific surgical risks should be considered in the context of established DBS ethical guidelines. Clinicians and researchers must develop strategies to challenge misperceptions about elevated surgical risk when appropriate and identify strategies to mitigate barriers to inclusion in DBS clinical trials. Our findings are encouraging for DBS clinical trials, showing that subjects with SZ or SAD undergoing surgical procedures have reasonable levels of postsurgical morbidity and mortality after surgical procedures. The established DBS guidelines for determining appropriate and safe surgical candidacy in treatment-refractory PD include adherence to pharmacological and medical treatments and consideration of individuals' surgical risk factors (21, 68). These established DBS guidelines can and should be reasonably applied to subjects with treatment-refractory SZ/SAD.

### Study limitations

The main limitation to our approach is that, given the nature of this study, we were unable to independently verify diagnoses, and ICD-10 and CPT codes were used as proxies for diagnoses and procedures. In one study, ICD-10 codes correctly identified 85% of schizophrenia cases (69). Misdiagnoses for SZ/SAD may have included cases with bipolar disorder with psychotic features, schizophreniform disorder, drug/medication-induced psychosis, other etiological medical conditions with psychotic symptoms, other psychotic disorders, delusional disorder, schizotypal personality disorders, brief psychotic disorder, or anti-NMDA receptor encephalitis, central nervous system damage, or epilepsy. In one study, ICD-9 codes correctly identified 75.6% of PD cases (70). PD may have mistakenly included some cases of multiple system atrophy, essential tremor, normal pressure hydrocephalus, dementia with Lewy bodies, corticobasal syndrome, or progressive supranuclear palsy. The diagnostic codes used for SZ/SAD, PD, and several other diagnoses did not include measures of severity.

Mortality and surgical morbidity outcomes were temporally linked to the surgical events but may be independent events. Some factors considered in this analysis to be postsurgical morbidity events (such as noncompliance with medical treatment and regimen) may or may not have influenced health outcomes. Some relevant diagnostic codes may have been missed by our approach explained in the methods (Table 3). Additionally, outcomes not evaluated in this study may be relevant, such as target-specific stimulation side-effects for new DBS indications. TriNetX does not accurately count events occurring <10 times; due to the low frequency of postoperative mortality after 1 month, we analyzed all surgical procedures, not just neurosurgical procedures that comprise <2% of matched PD and SZ/SAD cases (Figure 2B). While surgical decisions must be made on a case-by-case basis, results from this large data set are reassuring and will hopefully contribute to reduction of disparities in access to DBS clinical trials for new indications.

The focus of this research is on nonmaleficence. Other key ethical considerations that could not be investigated here directly but are particularly relevant to vulnerable individuals with severe symptoms of SZ/SAD include respect for persons. It is important that patients have capacity to make an informed decision when consenting to a DBS clinical trial and autonomy needs to be respected. Competent patients have the right to choose to participate or not participate in a clinical trial. Therefore, there is an obligation to educate even subjects with severe disabling symptoms who retain capacity about therapeutic opportunities so that they can make informed decisions for themselves.

## Conclusions

The findings of lower postsurgical mortality in subjects with SZ/SAD compared to PD demonstrate that the established ethical standards and guidelines for assessing capacity to consent and individual surgical risk may be reasonably applied to DBS clinical trials for subjects with SZ or SAD and the other new indications examined in this study. Even though stimulation risks and benefits can only be estimated with DBS clinical trials for new indications and brain targets, these relative surgical outcomes can be used to challenge misperceptions of elevated surgical risk and decrease disorder-specific disparities in access to advanced therapeutics that require neurosurgery.

## Data Availability

The original contributions presented in the study are included in the article/Supplementary Material, further inquiries can be directed to the corresponding author.
